# Randomized placebo-controlled study of intravenous methylnaltrexone in postoperative ileus

**DOI:** 10.3109/21556660.2013.838169

**Published:** 2013-08-27

**Authors:** Eugene R. Viscusi, James P. Rathmell, Alessandro Fichera, Sander R. Binderow, Robert J. Israel, Frank L. Galasso, Darryl Penenberg, Tong J. Gan

**Affiliations:** 1 Thomas Jefferson University, Philadelphia, PAUSA; 2 Massachusetts General Hospital and Harvard Medical School, Boston, MAUSA; 3 University of Chicago, Chicago, ILUSA; 4 Atlanta Colon and Rectal Surgery, Atlanta, GAUSA; 5 Progenics Pharmaceuticals, Inc., Tarrytown, NYUSA; 6 Duke University Medical Center, Durham, NCUSA

**Keywords:** Methylnaltrexone, Postoperative ileus, Opioids, Mu-opioid receptor antagonist

## Abstract

**Objective:**

This phase 2 study evaluated the safety and activity of intravenous methylnaltrexone on the duration of postoperative ileus in patients undergoing segmental colectomy.

**Methods:**

Adults (aged 18 years or older) with American Society of Anesthesiologists physical status of I, II, or III who underwent segmental colectomy, including partial colectomy, sigmoidectomy, cecectomy, or anterior proctosigmoidectomy, via laparotomy with general anesthesia, received intravenous methylnaltrexone 0.30 mg/kg or placebo every 6 h beginning within 90 min after end of surgery. Treatment continued until 24 h after the patient tolerated solid foods, was discharged, or for 7 d maximum. Efficacy endpoints included measures of gastrointestinal recovery and time to discharge eligibility.

**Results:**

A total of 65 patients (methylnaltrexone, *n* = 33; placebo, *n* = 32) were randomized. Mean time to first bowel movement was accelerated by 20 h (*p* = 0.038) and time to discharge eligibility was accelerated by 33 h (*p* = 0.049) with methylnaltrexone vs placebo. Opioid use was similar between groups until postoperative day 4, then fluctuated in the placebo group. Methylnaltrexone was generally well tolerated.

**Conclusions:**

In this study, intravenous methylnaltrexone significantly decreased time to postoperative bowel recovery and eligibility for hospital discharge by ∼1 d, with an adverse event profile similar to placebo. These were two of several exploratory endpoints; not all efficacy endpoints showed a significant difference between methylnaltrexone and placebo. The efficacy results in this trial were not seen in two subsequent large-scale studies.

## Introduction

Postoperative ileus (POI), a multi-factorial disorder, is commonly the result of gastrointestinal (GI) surgery1. Surgical manipulation of the bowel leads to POI1,2. It has been theorized that endogenous stimulation of opioid receptors can contribute to this neurally mediated reflex3–5. POI affects the entire GI tract1,6 and is associated with prolonged hospital stays at an estimated cost of more than $1 billion annually7.

Most patients who undergo major bowel surgery have moderate-to-severe pain requiring opioid analgesics. Opioids exacerbate and prolong POI by binding to µ-opioid receptors in the gut, resulting in decreased propulsive peristaltic waves and delayed intestinal transit1,2,6,8,9. Conventional treatments, including nasogastric suction, prokinetic agents, early enteral feeding, and the use of minimally invasive surgical techniques, have not been proven effective in preventing or alleviating POI1. A pharmacologic agent that selectively antagonizes the peripheral binding of opioids in the intestine while preserving central analgesia may be a useful treatment for POI. Peripheral µ-opioid receptor antagonists show promise in meeting these needs.

**Figure 1.**
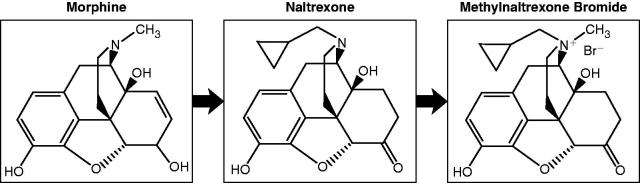
Chemical structures of morphine, the non-selective opioid antagonist naltrexone, and the selective opioid antagonist methylnaltrexone.

Naloxone has been shown to effectively reverse opioid-induced constipation10–12. However, because it crosses the blood–brain barrier, it also reverses the analgesic effect of opioid treatments and produces withdrawal symptoms2,13. Methylnaltrexone, a peripheral µ-opioid receptor antagonist, is a quaternary derivative of naltrexone (Figure 1). Methylnaltrexone has restricted access to the blood–brain barrier as a result of this modified structure9,14–16 and, thus, preserves central analgesia while reducing GI effects1,9,13. Subcutaneous methylnaltrexone is approved by the US Food and Drug Administration for the treatment of opioid-induced constipation in patients with advanced illness who are receiving palliative care, when response to laxative therapy has been insufficient17. Studies have demonstrated rapid laxation compared with placebo in patients with advanced illness and opioid-induced constipation18.

The present study was conducted to assess the safety and activity of intravenous (IV) methylnaltrexone used to shorten the duration of POI in patients who underwent open segmental colectomy.

## Patients and methods

This multi-center randomized, placebo-controlled phase 2 study was conducted in hospitals from July 2003 to December 2004. The protocol was approved by the ethics or institutional review board at each site and the study was conducted in accordance with the International Conference on Harmonization Good Clinical Practice Guidelines and the Declaration of Helsinki. Written informed consent was obtained from each patient prior to participation. Adult patients (18 years of age or older) with an American Society of Anesthesiologists physical status of I, II, or III who had undergone segmental colectomy—including partial colectomy, sigmoidectomy, cecectomy, or anterior proctosigmoidectomy—via laparotomy with general anesthesia were enrolled. Women of childbearing age were required to have negative pregnancy tests and use reliable birth control. All patients had received a standardized general anesthetic and used patient-controlled IV opioids for postoperative pain relief.

Patients were excluded if they had undergone surgery for complications related to inflammatory bowel disease, had recent abdominal radiation therapy, had surgery-related ostomies, or had received spinal or epidural anesthesia or analgesia. Other exclusion criteria included sensitivity to study medication or opioids, treatment with an investigational drug within the previous 30 d, prior treatment with vinca alkaloids, or significant medical history that may complicate the postoperative course, including a prolonged QTc interval.

This was a hypothesis-generating study; therefore, there was no pre-defined primary efficacy endpoint. All efficacy variables were considered exploratory. Efficacy was evaluated 4-times daily, regardless of study drug administration, using the gastrointestinal assessments listed in Table 1. In addition, time to first micturation after removal of the urinary catheter, time to administration of rescue antiemetics, and the amount of morphine-equivalent19–26 opioid medications administered were evaluated.

**Table 1.** Gastrointestinal efficacy measures.

• Time to tolerance of solid foods: no nausea or vomiting within 60 min of a second consecutive solid meal. The time to the endpoint was recorded as the time of the conclusion of the first solid meal. Meals had to be consumed within 2 h and the patient was required to eat >50% of the solid food
• Time to first bowel movement
• Time to discharge eligibility: tolerating solid foods, having had at least one bowel movement, normal body temperature, and no major complications (patients may have been discharged before reaching discharge eligibility)
• Time to solids-in, solids-out: toleration of solids and first bowel movement
• Time to actual discharge
• Time to first passage of flatus
• Time to gastrointestinal recovery: toleration of solids and first bowel movement or flatus
• Time to first tolerance liquid: 30 mL liquid by mouth with no nausea or vomiting within first 60 min of challenge
• Time to first tolerance of full liquids: >500 mL at the meal following tolerance of first 30 mL liquid with no nausea or vomiting within first hour of conclusion of full liquids

### Study design

Patients were randomized within 24 h prior to surgery to receive either IV methylnaltrexone (0.30 mg/kg in 50 mL of saline) or matching saline placebo allocated in a 1:1 ratio according to a randomization schedule prepared by Progenics Pharmaceuticals, Inc. that was provided to the investigational pharmacist at each study site. As each patient was enrolled in the study, the pharmacist opened the next sealed, sequentially numbered envelope containing the name of the study medication assigned to that patient. Only the pharmacist was unblinded at each site; the patients and investigators remained blinded during the study. The dose of 0.3 mg/kg methylnaltrexone was selected based on previous studies15,16. The first dose of study drug was administered within 90 min after the end of the surgical procedure. Study drug was administered intravenously over 20 min every 6 h until the patient could tolerate solid foods for 24 h, until the patient was discharged from the hospital, or until the patient received treatment for a maximum of 7 d.

Blood pressure and pulse were monitored prior to, at the end, and at 20, 60, 120, and 180 min after the first and second study drug infusions. For subsequent doses, they were monitored prior to and 20 min after dosing. Prior to and 60 min after the first study drug administration, then 4-times daily, patients were not only asked to assess their nausea, abdominal cramping, pain, and itching levels based on a verbal numeric scale (0–10 scale) but to also report on whether or not they had vomited. A 12-lead resting electrocardiogram (ECG) reading, physical examination findings and laboratory assessments (complete blood count and comprehensive metabolic panels) were obtained at the screening visit and at the end of treatment. Review of all concomitant medications and adverse event (AE) evaluations were performed at the beginning of and throughout the study. Patients were also monitored for any adverse effects at the follow-up 30 d after the last dose of study drug.

### Statistical analysis

Efficacy analyses used the intent-to-treat (ITT) population. The ITT population included all randomized patients who received at least one dose of study drug. Patients who discontinued treatment prematurely continued to be followed for efficacy through the end of the study. The primary objective was to assess the activity of methylnaltrexone on the duration of POI. A sample size of 30 patients per group was estimated to have ∼80% power to detect a minimum of 30 percentage points difference in the proportion of responders for a given endpoint at the 5% level of significance. Continuous endpoints were summarized by treatment group/study day and during the entire postoperative period and analyzed using a Wilcoxon signed-rank test. Categorical variables were tested using Pearson’s *χ*^2^ test for contingency tables. The distributions of efficacy event times were estimated using product limit (Kaplan-Meier) methods and compared between treatment groups by the 2-sided log-rank test. All statistical comparisons were performed at the 0.05 level of statistical significance without multiplicity adjustments.

All patients who received at least one dose of study drug were included in the analysis of the safety data. The number/percentage of patients experiencing one or more AEs or with any specific AE occurring in more than 5% of patients was tabulated.

## Results

**Figure 2.**
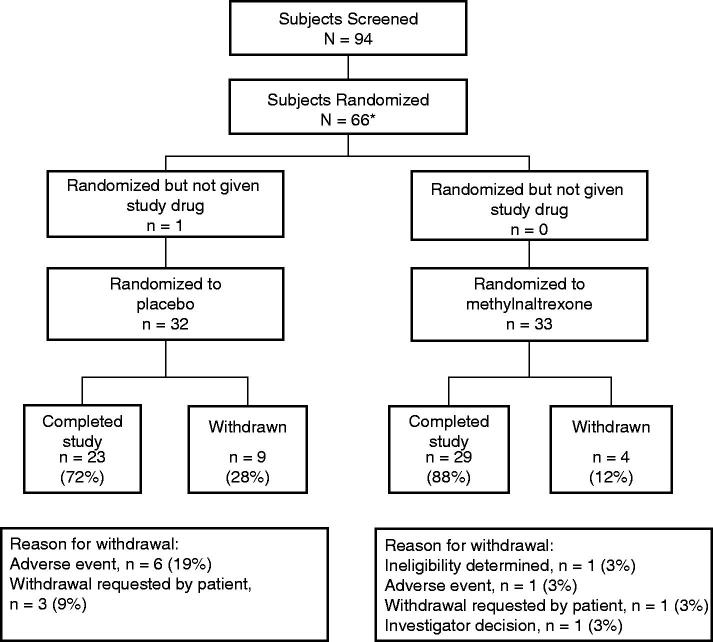
Patient disposition. *One ineligible patient was randomized to placebo but drug was not dispensed.

Of the 94 patients screened, 65 patients (33 in the methylnaltrexone group and 32 in the placebo group) were randomized and treated after surgery (Figure 2). Of these, 52 patients (29 in the methylnaltrexone group and 23 in the placebo group) completed treatment. Of the four patients in the methylnaltrexone group who discontinued treatment, one discontinued because of nausea, one was determined to be ineligible, one was withdrawn because of an investigator’s decision, and one patient withdrew on request. In the placebo group, nine patients discontinued, three because of nausea, one because of urticaria, one owing to an abdominal hematoma, one because of pneumonia, and three who withdrew consent.

The demographic and baseline characteristics of the patients were similar between the treatment groups (Table 2). The most common surgery type overall was sigmoid resection, and analyses of this patient sub-group showed similar trends of efficacy results to that of the entire population.

**Table 2.** Baseline patient demographics*.

Characteristic	Methylnaltrexone (*n* = 33)	Placebo (*n* = 32)	Total (*n* = 65)
Gender, *n* (%)			
Male	12 (36)	14 (44)	26 (40)
Female	21 (64)	18 (56)	39 (60)
Race, *n* (%)			
White	27 (82)	26 (81)	53 (82)
Black	6 (18)	6 (19)	12 (18)
Mean age, years (SD)	58.7 (13.8)	54.8 (11.1)	56.8 (12.6)
Mean weight, kg (SD)	76.9 (14.0)	85.1 (19.5)	80.9 (17.3)
Type of surgery, *n* (%)			
Sigmoid resection	17 (51.5)	11 (34.4)	28 (43.1)
Right hemicolectomy	7 (21.2)	13 (40.6)	20 (30.8)
Left hemicolectomy	2 (6.1)	4 (12.5)	6 (9.2)
Low anterior resection	5 (15.2)	0	5 (7.7)
Colostomy takedown	0	3 (9.4)	3 (4.6)
Transverse colectomy	1 (3.0)	1 (3.1)	2 (3.1)
Total colectomy	1 (3.0)	0	1 (1.5)

*Intent-to-treat population.

### Efficacy

Overall, patients receiving methylnaltrexone had a more favorable response for most efficacy measures compared with patients receiving placebo (Table 3). The mean time to the first bowel movement was accelerated by 20 h in patients receiving methylnaltrexone (mean time 98.0 ± 5.7 h) compared with placebo (mean time 118.1 ± 10.3 h; *p* = 0.038 log-rank test), and time to discharge eligibility was accelerated by 33 h (mean times 116.1 ± 6.9 h for methylnaltrexone vs 148.7 ± 17.2 h for placebo; *p* = 0.049 log-rank test). Mean times to other measures of efficacy, including first tolerance of full liquids and first tolerance of solids, were accelerated among patients receiving methylnaltrexone compared with placebo but did not reach statistical significance. Only time to first tolerance of liquids was similar between the two treatment groups.

**Table 3.** Mean time to gastrointestinal measures of efficacy*.

Efficacy variables	Methylnaltrexone hours ± SD	Placebo hours ± SD	Difference hours	*p-*value^a^
First tolerance of solids	97.4 ± 11.6	123.9 ± 17.1	26.5	0.205
First bowel movement	98.0 ± 5.7	118.1 ± 10.3	20.1	0.038
Discharge eligibility	116.1 ± 6.9	148.7 ± 17.2	32.6	0.049
Solids-in, solids-out	124.1 ± 8.6	151.2 ± 15.6	27.2	0.124
Actual discharge	138.3 ± 6.7	164.5 ± 16.1	26.2	0.159
First passage of flatus	88.4 ± 6.5	97.8 ± 10.0	9.4	0.327
GI recovery^b^	114.8 ± 9.4	136.8 ± 15.9	22.0	0.263
First tolerance of liquids (30 mL)	55.3 ± 7.6	54.7 ± 9.2	−0.6	0.925
First tolerance of full liquids (500 mL)	68.1 ± 9.0	97.3 ± 18.7	29.2	0.119

*Intent-to-treat population. ^a^Log-rank *χ*^2^. ^b^Gastrointestinal recovery defined as toleration of solids and first bowel movement or flatus.

The mean time to first administration of rescue antiemetics was longer for patients in the methylnaltrexone group (75.7 ± 12.8 h) than for those in the placebo group (31.3 ± 5.3 h), although the difference was not statistically significant (*p* = 0.411 log-rank test). At 60 min after the first administration of study drug, 6% of patients in the methylnaltrexone group and 12% of those in the placebo group reported vomiting.

Opioid use was similar between the two treatment groups immediately after surgery and on postoperative days (PODs) 2 and 3 (Table 4). Opioid use steadily decreased from POD 1–4. Beyond POD 4, the levels of opioid use continued to decrease for the methylnaltrexone group for the rest of the study (up to day 8), but varied for the placebo group.

**Table 4.** Daily opioid* dose (mg) by treatment group.

	Methylnaltrexone		Placebo	
Postoperative day	*n*	Median (range)	*n*	Median (range)
1	32	33.4 (8.0–271.9)	32	49.6 (6.7–295.2)
2	31	29.5 (2.2–538.0)	32	39.5 (6.7–309.8)
3	31	29.3 (2.2–318.0)	30	31.1 (0–309.8)
4	27	11.5 (0–70.0)	27	23.3 (0–383.2)
5	18	9.9 (0–70.0)	16	0.8 (0–46.7)
6	12	8.8 (0–70.0)	10	3.7 (0–45.8)
7	10	0 (0–70.5)	7	23.3 (0–73.7)
8	8	0 (0–28.9)	4	4.7 (0–62.6)

*Intravenous morphine equivalent19–26.

Patient assessments of pain using the verbal numeric scale generally decreased from POD 1–7 in both treatment groups. The scores were similar between the two treatment groups at each time point. On Day 1 before dosing, pain scores were 6.33 and 6.67 in the methylnaltrexone and placebo groups, respectively. These scores decreased to 5.55 and 5.35 60 min post-dose, to 3.44 and 3.46 at the end of POD 1, and continued to decrease in a similar manner in both treatment groups through POD 7. There was little evidence of nausea, cramping, or itching.

### Safety

Methylnaltrexone was generally well tolerated (see Table 5 for a summary of AEs occurring in at least 10% of patients in either treatment group). Gastrointestinal AEs were reported more frequently in the placebo group compared with the methylnaltrexone group, including nausea (63% vs 30%, respectively), vomiting (25% vs 12%, respectively), and abdominal pain (13% vs 0%, respectively).

**Table 5.** Adverse events reported in ≥10% of patients in either treatment group.

Adverse event	Methylnaltrexone (*n* = 33), *n* (%)	Placebo (*n* = 32), *n* (%)
Pyrexia	11 (33)	12 (38)
Nausea	10 (30)	20 (63)
Hypotension	6 (18)	3 (9)
Pruritus	6 (18)	7 (22)
Hypomagnesemia	5 (15)	5 (16)
Urine output decreased	5 (15)	5 (16)
Hypocalcemia	4 (12)	3 (9)
Hypophosphatemia	4 (12)	4 (13)
Vomiting	4 (12)	8 (25)
Hypokalemia	3 (9)	5 (16)
Hypoxia	3 (9)	6 (19)
Hypertension	2 (6)	5 (16)
Postoperative ileus	2 (6)	5 (16)
Dyspnea	1 (3)	4 (13)
Wound infection	1 (3)	4 (13)
Abdominal pain	0 (0)	4 (13)
Headache	0 (0)	6 (19)

Symptoms associated with prolonged POI were reported as an AE by the investigators. The incidence of POI, reported as an AE, was reduced 2-fold in patients treated with methylnaltrexone compared with patients on placebo. POI, reported as an AE, occurred in five (16%) patients in the placebo group and in two (6%) patients in the methylnaltrexone group. Hypotension was the only AE notably more frequent in the methylnaltrexone group compared with the placebo group (18% vs 9%, respectively), and all occurrences were mild-to-moderate (grades 1 or 2 according to Common Toxicity Criteria, version 2.0) in severity and judged by the investigators as not related or unlikely to be related to study medication.

Adverse events possibly related to study drug occurred in three patients receiving methylnaltrexone (diarrhea, insomnia, and pruritus) and in two patients receiving placebo (nausea and hypotension). All were mild-to-moderate in severity. Serious AEs that occurred during the treatment and follow-up periods are reported in Table 6. All of the serious AEs resolved and none were judged to be related to study drug. No deaths occurred in this study.

**Table 6.** Serious adverse events.

Study period	Treatment group	Adverse events^a^
Treatment	Placebo	Enterocutaneous fistula, wound dehiscence
	Placebo	Pneumonia
Follow-up	Placebo	Ureteric obstruction
	Placebo	Enterocutaneous fistula, wound dehiscence
	Methylnaltrexone	Supraventricular tachycardia, ruptured resection, anastomotic leak, postoperative ileus, hyperkalemia
	Placebo	Pneumonia
	Placebo	Abdominal pain^b^

^a^Individual patients with adverse event. ^b^Abdominal pain was reported as three adverse events in this patient.

The clinically significant laboratory findings that occurred during the study included low hemoglobin, hematocrit, and red blood cells in eight patients, three of whom were in the placebo group and five in the methylnaltrexone group. All clinically significant laboratory abnormalities were attributed to an underlying disease. For vital signs and ECGs, the changes from baseline within the treatment group and the differences between treatment groups were relatively small, consistent with postoperative recovery in these patients, and not indicative of any clinically relevant effect of the investigational drug.

## Discussion

In this study, patients receiving methylnaltrexone had a more rapid recovery of bowel function than those receiving placebo.

Notably, the mean time to first bowel movement was accelerated by 20 h in patients treated with methylnaltrexone compared with those receiving placebo. Patients receiving methylnaltrexone also had shorter times to first tolerance of full liquids and solids compared with those receiving placebo, although the differences between groups did not reach statistical significance. The mean time to antiemetic use was longer for patients receiving methylnaltrexone compared with those receiving placebo, although this difference did not reach statistical significance. Many patients did not require antiemetics during the 7-day study period. The increased and earlier use of antiemetics among patients who received placebo is consistent with the increased incidence of GI AEs, such as nausea and vomiting, compared with patients who received methylnaltrexone. The use of individual endpoints of gastrointestinal function allow for a better characterization of the effect of methylnaltrexone on upper and lower bowel recovery. These results suggest that IV methylnaltrexone may improve postoperative recovery of both upper and lower GI functions.

Time to discharge eligibility was significantly shorter among patients receiving methylnaltrexone compared with placebo (*p* = 0.049 log-rank test). The difference between the treatment groups is clinically relevant and may have an added economic benefit of reducing post-surgical hospital stays. Time to discharge eligibility is a composite endpoint, defined as the ability to tolerate solid foods, passage of at least one bowel movement, normal body temperature, and the absence of major complications. Because actual discharge may be influenced by factors unrelated to POI or by factors unrelated to medical status, discharge eligibility is a more meaningful measure in this study.

Overall, methylnaltrexone was well tolerated. Patients receiving placebo were more likely to report GI AEs, as well as headache, hypoxia, hypertension, and dyspnea, compared with those receiving methylnaltrexone. Hypotension was the only AE reported notably more often among patients receiving methylnaltrexone compared with placebo. Pain, as assessed by patients using the verbal numeric scale, was similar and generally decreased over time in both treatment groups during the study. Opioid use was also similar between both treatment groups. The similarity in pain intensity rating and opioid use between the treatment groups supports the peripheral µ-opioid receptor action of methylnaltrexone and preservation of central µ-opioid receptor activity.

It is interesting to note that although nausea and vomiting were reported as AEs twice as often in the placebo group compared with methylnaltrexone, between-group differences were not evident on patient assessments recorded using the verbal numeric scale. The apparent inconsistency between the patient assessments and reported AEs may be explained by differences in how the data were captured. Whereas AEs were collected through spontaneous patient reports, direct questioning, laboratory assessments, and physical examination findings, the verbal numeric scale evaluated the level of nausea, vomiting, cramping, and itching specifically at the time of the assessment.

In this study, treatment with IV methylnaltrexone at a dose of 0.30 mg/kg administered every 6 h for up to 7 d in patients after segmental colectomy appeared to be well tolerated. The lower incidence of GI AEs in patients treated with methylnaltrexone compared with placebo is consistent with the activity of methylnaltrexone in shortening the time to recovery from POI.

Clear identification of the return of normal bowel function following GI surgery is a challenge. Typically, motility is believed to be restored within 24 h after surgery in the stomach and small bowel and within 48–72 h in the colon27,28. However, there is no standard definition of persistent ileus, the typical period for ileus to last, or endpoints to determine recovery of bowel function7. Endpoints that are typically used, such as time to first tolerance of food or time to first flatus, are based on studies involving small numbers of patients. Further complicating the selection of endpoints for GI recovery is the recurrence of POI more than a day after it has resolved7. The specific GI efficacy endpoints used in our study are intended to provide a broad characterization of complete GI recovery.

These current findings are consistent with another µ-opioid receptor antagonist, alvimopan, which is approved in the US for acceleration of the time to upper and lower GI recovery following partial large or small bowel resection surgery with primary anastomosis29. In clinical trials, alvimopan exhibited advantages in some GI recovery endpoints while failing to reach significance in others30–32. In contrast, results of two phase 3 studies of IV methylnaltrexone, each with ∼500 patients undergoing open segmental colectomy, failed to show an improvement in time to first bowel movement and in other GI recovery endpoints33. The reasons for these findings are unclear, although issues related to the dose, timing of the first dose, and choice of primary outcome measure have been discussed previously33.

## Conclusions

In this phase 2 study, IV methylnaltrexone significantly decreased the time to postoperative bowel recovery, as evidenced by a reduction in time to first bowel movement and discharge eligibility. Additional research is necessary to further assess the conditions under which methylnaltrexone may show benefit in patients with postoperative ileus.

## Transparency

### Declaration of funding

This study was sponsored by Progenics Pharmaceuticals Inc., which has a proprietary commercial interest in methylnaltrexone. Funding for this manuscript was provided by Wyeth Research, which was acquired by Pfizer Inc. in October 2009. No author received an honorarium or other form of financial support related to the development of this manuscript.

### Declaration of financial/other relationships

EV is a consultant and advisor for Salix Pharmaceuticals, Inc. JR, SB, AF, and DP have nothing to disclose. RI is an employee of Progenics Pharmaceuticals, which has a financial interest in Relistor. FG is a consultant for Progenics Pharmaceuticals. TG has received grants/research funding from Cubist Pharmaceuticals Inc., Purdue Pharma, LP.

## Acknowledgments

The authors thank Cheryl Jones of On Assignment Clinical Research, who was a paid consultant to Pfizer in connection with the development of this manuscript. Editorial support was provided by John Simmons, MD, of Peloton Advantage and was funded by Pfizer. Editorial support was also provided by Andrew Barrett, PhD, of Salix Pharmaceuticals. Presented in part at the 2007 Gastrointestinal Cancers Symposium of the American Society of Clinical Oncology, January 19–21, 2007, in Orlando, FL. ClinicalTrials.gov identifier number: NCT00387309

## References

[1] Kehlet H Holte K Review of postoperative ileus. Am J Surg 2001;182(5A Suppl):3S–10S 11755891 10.1016/s0002-9610(01)00781-4

[2] Taguchi A Sharma N Saleem RM et al Selective postoperative inhibition of gastrointestinal opioid receptors. N Engl J Med 2001;345:935–40 11575284 10.1056/NEJMoa010564

[3] Brix-Christensen V Tonnesen E Sanchez RG et al Endogenous morphine levels increase following cardiac surgery as part of the antiinflammatory response? Int J Cardiol 1997;62:191–7 9476677 10.1016/s0167-5273(97)00229-5

[4] Yoshida S Ohta J Yamasaki K et al Effect of surgical stress on endogenous morphine and cytokine levels in the plasma after laparoscopic or open cholecystectomy. Surg Endosc 2000;14:137–40 10656946 10.1007/s004649900085

[5] Sanger GJ Tuladhar BR The role of endogenous opioids in the control of gastrointestinal motility: predictions from *in vitro* modelling. Neurogastroenterol Motil 2004;16(Suppl 2):38–45 15357850 10.1111/j.1743-3150.2004.00556.x

[6] Fukuda H Suenaga K Tsuchida D et al The selective mu opioid receptor antagonist, alvimopan, improves delayed GI transit of postoperative ileus in rats. Brain Res 2006;1102:63–70 16797494 10.1016/j.brainres.2006.02.092

[7] Delaney CP Clinical perspective on postoperative ileus and the effect of opiates. Neurogastroenterol Motil 2004;16(Suppl 2):61–6 15357853 10.1111/j.1743-3150.2004.00559.x

[8] Manara L Bianchi G Ferretti P et al Inhibition of gastrointestinal transit by morphine in rats results primarily from direct drug action on gut opioid sites. J Pharmacol Exp Ther 1986;237:945–9 3012075

[9] Yuan CS Foss JF O'Connor M et al Methylnaltrexone prevents morphine-induced delay in oral-cecal transit time without affecting analgesia: a double-blind randomized placebo-controlled trial. Clin Pharmacol Ther 1996;59:469–75 8612393 10.1016/S0009-9236(96)90117-4

[10] Culpepper-Morgan JA Inturrisi CE Portenoy RK et al Treatment of opioid-induced constipation with oral naloxone: a pilot study. Clin Pharmacol Ther 1992;52:90–5 1623695 10.1038/clpt.1992.106

[11] De Schepper HU Cremonini F Park MI et al Opioids and the gut: pharmacology and current clinical experience. Neurogastroenterol Motil 2004;16:383–94 15305992 10.1111/j.1365-2982.2004.00513.x

[12] Latasch L Zimmermann M Eberhardt B et al Treatment of morphine-induced constipation with oral naloxone [German]. Anaesthesist 1997;46:191–4 9163262 10.1007/s001010050390

[13] Sinatra RS Peripherally acting mu-opioid-receptor antagonists and the connection between postoperative ileus and pain management: the anesthesiologist's view and beyond. J Perianesth Nurs 2006;21(2A Suppl):S16–23 16597531 10.1016/j.jopan.2006.01.016

[14] Foss JF O'Connor MF Yuan CS et al Safety and tolerance of methylnaltrexone in healthy humans: a randomized, placebo-controlled, intravenous, ascending-dose, pharmacokinetic study. J Clin Pharmacol 1997;37:25–30 9048269 10.1177/009127009703700105

[15] Yuan CS Foss JF O'Connor M et al Methylnaltrexone for reversal of constipation due to chronic methadone use: a randomized controlled trial. JAMA 2000;283:367–72 10647800 10.1001/jama.283.3.367

[16] Yuan CS Wei G Foss JF et al Effects of subcutaneous methylnaltrexone on morphine-induced peripherally mediated side effects: a double-blind randomized placebo-controlled trial. J Pharmacol Exp Ther 2002;300:118–23 11752106 10.1124/jpet.300.1.118

[17] Relistor [package insert]. Raleigh, NC, and Tarrytown, NY: Salix Pharmaceuticals and Progenics Pharmaceuticals, 2012

[18] Thomas J Karver S Cooney GA et al Methylnaltrexone for opioid-induced constipation in advanced illness. N Engl J Med 2008;358:2332–43 18509120 10.1056/NEJMoa0707377

[19] American Pain Society. Principles of analgesic use in the treatment of acute pain and cancer pain. 6th ed. Glenview, IL: American Pain Society, 2008.

[20] Duragesic [package insert]. Titusville, NJ: Janssen Pharmaceuticals, Inc., 2012

[21] Foley KM The treatment of cancer pain. N Engl J Med 1985;313:84–95 2582259 10.1056/NEJM198507113130205

[22] Goodman SR Kim-Lo SH Ciliberto CF et al Epinephrine is not a useful addition to intrathecal fentanyl or fentanyl-bupivacaine for labor analgesia. Reg Anesth Pain Med 2002;27:374–9 12132061 10.1053/rapm.2002.33283

[23] Lehmann KA Patient-controlled analgesia with opioids. In: Stein C, Ed. Opioids in Pain control: basic and clinical aspects. Cambridge, UK: Cambridge University Press, 1998 pp. 270–94

[24] Lichtor JL Sevarino FB Joshi GP et al The relative potency of oral transmucosal fentanyl citrate compared with intravenous morphine in the treatment of moderate to severe postoperative pain. Anesth Analg 1999;89:732–8 10475315 10.1097/00000539-199909000-00038

[25] OxyContin [package insert]. Stamford, CT: Purdue Pharma, 2012

[26] VA/DoD clinical practice guideline for the management of opioid therapy for chronic pain. Washington, DC: Veterans Health Administration, Department of Defense, 2003. http://health.utah.gov/prescription/pdf/guidelines/VA_DoD%20clinical%20practice%20guideline%20for%20the%20management%20of%20opioid%20therapy%20for%20chronic%20pain.pdf. Accessed April 16, 2013.

[27] Steinbrook RA An opioid antagonist for postoperative ileus. N Engl J Med 2001;345:988–9 11575292 10.1056/NEJM200109273451309

[28] Woods JH Erickson LW Condon RE et al Postoperative ileus: a colonic problem? Surgery 1978;84:527–33 99829

[29] Entereg [package insert]. Research Triangle Park, NC: GlaxoSmithKline, 2009

[30] Wolff BG Michelassi F Gerkin TM et al Alvimopan, a novel, peripherally acting mu opioid antagonist: results of a multicenter, randomized, double-blind, placebo-controlled, phase III trial of a major abdominal surgery and postoperative ileus. Ann Surg 2004;240:728–34 15383800 10.1097/01.sla.0000141158.27977.66 PMC1356474

[31] Wolff BG Viscusi ER Delaney CP et al Patterns of gastrointestinal recovery after bowel resection and total abdominal hysterectomy: pooled results from the placebo arms of alvimopan phase III North American clinical trials. J Am Coll Surg 2007;205:43–51 17617331 10.1016/j.jamcollsurg.2007.02.026

[32] Wolff BG Weese JL Ludwig KA et al Postoperative ileus-related morbidity profile in patients treated with alvimopan after bowel resection. J Am Coll Surg 2007;204:609–16 17382220 10.1016/j.jamcollsurg.2007.01.041

[33] Yu CS Chun HK Stambler N et al Safety and efficacy of Methylnaltrexone in shortening the duration of postoperative ileus following segmental colectomy: results of two randomized, placebo-controlled Phase 3 trials. Dis Colon Rectum 2011;54:570–8 21471758 10.1007/DCR.0b013e3182092bde

